# Carbon monoxide releasing molecule-A1 improves nonalcoholic steatohepatitis via Nrf2 activation mediated improvement in oxidative stress and mitochondrial function

**DOI:** 10.1016/j.redox.2019.101314

**Published:** 2019-08-31

**Authors:** Kapil K. Upadhyay, Ravirajsinh N. Jadeja, Hitarthi S. Vyas, Bhaumik Pandya, Apeksha Joshi, Aliasgar Vohra, Menaka C. Thounaojam, Pamela M. Martin, Manuela Bartoli, Ranjitsinh V. Devkar

**Affiliations:** a Metabolic Endocrinology Division, Department of Zoology, Faculty of Science, The Maharaja Sayajirao University of Baroda, Vadodara, Gujarat, 390002, India; b Department of Biochemistry and Molecular Biology, Augusta University, Augusta, GA, 30912, USA; c Georgia Cancer Center, Augusta University, Augusta, GA, 30912, USA; d Department of Ophthalmology, Medical College of Georgia, Augusta University, Augusta, GA, 30912, USA

**Keywords:** CORM-A1, NASH, Nrf2, Mitochondria, ROS

## Abstract

Nuclear factor-erythroid 2 related factor 2 (Nrf2)-mediated signaling plays a central role in maintaining cellular redox homeostasis of hepatic cells. Carbon monoxide releasing molecule-A1 (CORM-A1) has been reported to stimulate up-regulation and nuclear translocation of Nrf2 in hepatocytes. However, the role of CORM-A1 in improving lipid metabolism, antioxidant signaling and mitochondrial functions in nonalcoholic steatohepatitis (NASH) is unknown. In this study, we report that CORM-A1 prevents hepatic steatosis in high fat high fructose (HFHF) diet fed C57BL/6J mice, used as model of NASH. The beneficial effects of CORM-A1 in HFHF fed mice was associated with improved lipid homeostasis, Nrf2 activation, upregulation of antioxidant responsive (ARE) genes and increased ATP production. As, mitochondria are intracellular source of reactive oxygen species (ROS) and important sites of lipid metabolism, we further investigated the mechanisms of action of CORM-A1-mediated improvement in mitochondrial function in palmitic acid (PA) treated HepG2 cells. Cellular oxidative stress and cell viability were found to be improved in PA + CORM-A1 treated cells via Nrf2 translocation and activation of cytoprotective genes. Furthermore, in PA treated cells, CORM-A1 improved mitochondrial oxidative stress, membrane potential and rescued mitochondrial biogenesis thru upregulation of Drp1, TFAM, PGC-1α and NRF-1 genes. CORM-A1 treatment improved cellular status by lowering glycolytic respiration and maximizing OCR. Improvement in mitochondrial respiration and increment in ATP production in PA + CORM-A1 treated cells further corroborate our findings. In summary, our data demonstrate for the first time that CORM-A1 ameliorates tissue damage in steatotic liver via Nrf2 activation and improved mitochondrial function, thus, suggesting the anti-NASH potential of CORM-A1.

## Highlights

• CORM-A1 facilitates Nrf2 translocation and regulates cellular redox homeostasis in liver.
• CORM-A1 improves antioxidant status and lipid metabolism in liver.
• CORM-A1 induces mitochondrial biogenesis, improves energetics and cellular respiration in HepG2 cells.

## Graphical abstract

**Figure:**
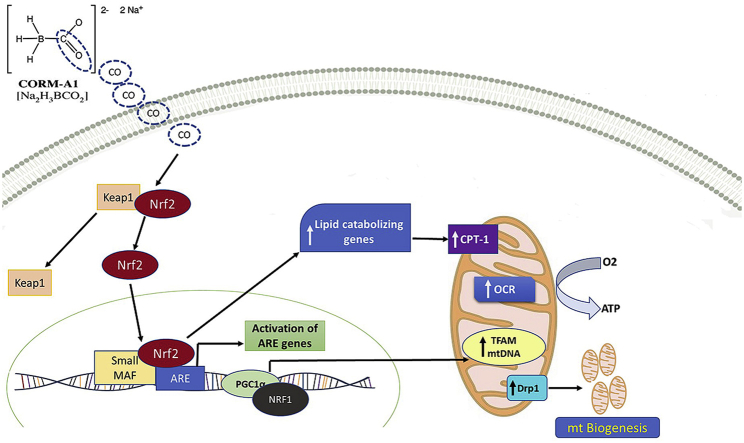

## 1 Introduction

Multitude of metabolic diseases, including non-alcoholic steatohepatitis (NASH), have been implicated to higher consumption of fat-rich and high calorie foods [1]. About 15% of the total obese individuals with symptoms of metabolic syndrome constitute the high-risk group for NASH. Ethnicity, dietary habits, genetic and environmental factors further contribute towards the observed variations in occurrence of NASH [2,3]. Excess lipid accumulation in hepatocytes, high oxidative stress and inflammation are the key players in pathogenesis of NASH [4]. Currently used symptomatic treatment protocols for NASH include the lipid lowering, anti-diabetic, antioxidants or anti-inflammatory drugs coupled with changes in lifestyle. However, no FDA approved drug is presently available for this potentially lethal disease [5]. Patients with NASH develop anomalies in the ultrastructure of mitochondria, impairment of hepatic ATP synthesis and increased mitochondrial ROS production [6,7]. Lipid peroxidation, cytokine production and fatty manifestations in liver causes cell death and overall impairment of liver function [8].

The transcription factor nuclear factor erythroid 2-related factor 2 (Nrf2) enables cell survival and adaptation under conditions of stress by regulating cytoprotective proteins, intracellular antioxidants, anti-inflammatory and detoxifying enzymes. In addition, Nrf2 also protects the liver against steatosis by restraining lipogenesis and by improving oxidation of unsaturated fats [9]. Therefore, a variety of Nrf2 activators such as phytochemicals, drugs or gasotransmitters have been investigated for their therapeutic potential in treating metabolic disorders [[10], [11], [12]].

Gasotransmitters (CO, H_2_S and NO) are molecules that are naturally produced and degraded within the human body. In liver, carbon monoxide (CO) is produced endogenously by heme oxygenase (HO)-mediated degradation of heme. This process also yields free iron and biliverdin that is rapidly converted into the antioxidant bilirubin [13]. Unlike other gasotransmitters, CO is relatively stable and has affinity towards transitional metals [14]. High levels of CO have been detected in breath of asthmatic and diabetic patients that underwent decrement following steroid or insulin treatment [15,16]. CO has been implicated in regulating mitochondrial ROS, cytochrome c oxidase, oxygen consumption, oxidative metabolism and overall mitochondrial functioning [17]. CO is also known to have beneficial effects against inflammation and hemorrhagic shock [18,19]. CO-releasing molecules (CO-RMs), are classes of organometallic compounds that have been developed to mimic the antioxidant, anti-inflammatory and cytoprotective properties of CO by ensuring its sustained release in biological systems [20]. Carbon monoxide releasing molecule A-1 (CORM-A1) has a boron core and is reported for slow and sustained release of CO in living systems (t_1/2_ = 21 min) [21]. Therapeutic effects of CORM-A1 in various diseases model such as diabetes, myocardial infarction and posterior uveitis is well established [[22], [23], [24]].

Reported attributes of CORM-A1 in alleviating oxidative stress and providing cytoprotection forms the basis of our hypothesis. Previous studies conducted in our lab had shown that CORM-A1 reduces oxidative stress in acetaminophen induced liver injury in mice via Nrf2 activation [25]. In this study, we had used *in vivo* and *in vitro* experimental models to evaluate the effects of CORM-A1 in improving various features associated with pathology of NASH i.e. hepatic steatosis, oxidative stress, inflammation and mitochondrial dysfunction.

## 2 Materials and methods

### 2.1 Chemical and reagents

Chemicals for cell culture like Dulbecco's modified eagle's medium (DMEM), fetal bovine serum (FBS), trypsin phosphate versene glucose (TPVG), bovine serum albumin (BSA) and antibiotic-antimycotic solution were purchased from Hi-media laboratories (Mumbai, India). TRIzol and SYBR select master mix were procured from Invitrogen (CA, USA). iScript cDNA synthesis kit was procured from Bio-Rad (CA, USA). Antibodies Nrf2 (12721S), HO-1 (70081S), β-actin (4970S) and 2° Antibody (7074P2) were purchased from cell signaling technology (Danvers, MA). PGC-1α (ab54481), Keap1 (ab139729), NRF-1 (ab175932) were purchased from Abcam (Cambridge, MA, USA). RNA-later stabilizing solution was purchased from Ambion Inc. (USA). CORM-A1, haematoxylin, eosin and palmitic acid (PA) were purchased from Sigma aldrich (St. Louis, MO, USA). Methanol, dimethyl sulphoxide (DMSO), 3-(4,5-dimethylthiazol-2-yl)-2,5-diphenyl tetrazolium bromide (MTT) were purchased from Sisco research laboratory Pvt. Ltd. (Mumbai, India).

### 2.2 Animal studies and experimental protocols

C57BL/6J male mice (6–8 weeks of age) were purchased from ACTREC Mumbai and maintained as per CPCSEA standard guidelines (23 ± 2 °C, LD 12:12, laboratory chow and water ad libitum) followed by a week-long acclimatization. Protocol was approved by Institutional Animal Ethical Committee (IAEC) (Approval no. MSU-Z/IAEC/02-2017) and experiments were conducted in CPCSEA approved animal house facility of Department of Zoology, The Maharaja Sayajirao University of Baroda, Vadodara, Gujarat, India (827/GO/Re/S/04/CPCSEA).

Mice were randomly divided into three groups with six animals per group. The entire period of experiment was of 16 weeks. Group I (SD) was fed with standard diet (SD). Group II (HFHF) was fed with high fat diet + 20% Fructose (HFHF diet) [26]. Group III (HFHF + CORM-A1) was fed with HFHF diet for 16 weeks and CORM-A1 was injected (ip:2 mg/kg/day) from 9th to 16th week. Food intake, water intake and body weights were recorded every week throughout the period of study. At the end of 16 weeks, animals were fasted overnight and whole blood was collected by retro-orbital sinus puncture under mild isoflurane anaesthesia. Whole blood was centrifuged (at 4 °C and 3000 rpm for 10 min) and serum was collected and stored. Later, mice were sacrificed, and liver and visceral fat were collected. These tissue samples were stored in 10% formalin (for histopathology), in RNAlater (for gene expression studies) or at −80°C (for protein analysis).

### 2.3 Serum biochemical parameters

Levels of circulating enzymes indicative of liver function (AST, ALT and ALP) and serum lipid profile (TL, TC, TG, LDL, VLDL, CHL/HDL and LDL/HDL ratio) were estimated using commercially available kits (Reckon Diagnostic kits, Vadodara, Gujarat, India).

### 2.4 Liver histopathology

Formalin fixed liver and adipose tissue (n = 6) were dehydrated and embedded in paraffin wax blocks and cut into 5  μm thick sections. These sections were stained with haematoxylin and eosin (H&E) and were observed and photographed (Leica DM 2500 microscope). Investigators blinded to this study conducted scoring of ballooning hepatocytes and steatotic liver sections of control and treated mice [27]. Adipose tissue sections were observed, photographed and morphometric scoring was done for the same.

### 2.5 Quantitative real-time polymerase chain reaction (qPCR) analysis

Total RNA content from control and treated liver and HepG2 cells was isolated using TRIzol reagent and cDNA was synthesized using iScript cDNA Synthesis kit (BIO-RAD CA, USA). mRNA levels of candidate genes (S. Table- 1) were quantified by qPCR analysis (QuantStudio-3 real time PCR, Life Technologies, CA, USA) using SYBR Select Master Mix. The data were normalized to the internal control GAPDH and analysed using 2^−ΔΔCT^ method.

### 2.6 Immunoblot analysis

Control and treated liver samples and HepG2 cells were homogenized with ice-cold lysis buffer. Nuclear proteins were isolated as described in NE-PER nuclear extraction kit (Thermo Scientific USA). Total protein content was quantified by Bradford assay wherein, equal amount (40 μg) was separated using 10% SDS polyacrylamide gel electrophoresis. Proteins were transferred on to PVDF membrane (Bio-Rad, USA) and primary antibodies for Nrf2, HO-1, Keap1, NRF-1 or PGC 1-α (1:1000) were added followed by secondary anti-rabbit horseradish peroxidase antibody (1:5000). Blots were stripped using stripping buffer (Thermo Scientific, Wilmington, DE) and re-probed with goat anti-rabbit Lamin B and β-actin antibody (1:5000) to determine equivalent loading. Blots were developed using ECL reagent (Bio-Rad, Hercules, CA) and visualized in iBright Imaging System.

### 2.7 Co-immunoprecipitation assay

The interaction of Nrf2 with Keap1 following CORM-A1 treatment to HepG2 cells was assayed by co-immunoprecipitation (co-IP) assay using magnetic beads according to the manufacturer's protocol (Pierce Classic Magnetic IP/Co-IP Kit). 500 μg of protein from different experimental groups was mixed with 10 μg Nrf2 antibody overnight at 4 °C to form the immune complex. 25 μl of pre-washed Pierce Protein A/G Magnetic Beads were placed into the above immune complex and incubated for 1 h with mixing. Then the beads were washed, and the target antigen was eluted with alternative elution method. Target antigen and the binding proteins were immunoblotted with the indicated antibodies by Western blot assay.

### 2.8 Mitochondrial DNA copy number

Mitochondrial DNA (mtDNA) was used to determine mitochondrial density using q-PCR. Briefly, total DNA was isolated from liver tissue or HepG2 cells using GeneJET genomic DNA purification kit (Thermo Scientific, USA) according to the manufacturer's instructions. The mtDNA copy number was calculated from the ratio of Cytochrome b (mitochondrial encoded gene) to Nuclear18s rRNA (nuclear-encoded gene).

### 2.9 Total ATP content

ATP content was determined using an ATP Determination Kit (A22066, Molecular Probes). Each reaction contained 1.25 μg/ml firefly luciferase, 0.5 mM D-luciferin and 1 mM dithiothreitol in 100 ml reaction buffer. At the end of each experiment, ATP content was measured in liver tissue. After 15-min incubation luminescence was measured in Synergy HTX Multimode Reader (Bio-Tek instruments, Inc., Winooski, VT). Results were expressed as arbitrary units of luminescence compared to that measured in SD group.

### 2.10 Cells culture and treatment

Human Hepatoma (HepG2) cells were procured from National Centre for Cell Science (NCCS, Pune, India). Cells were maintained in CO_2_ incubator (Thermo scientific, forma series II 3110, USA) at 37 °C and 5% CO_2_ in DMEM, supplemented with 10% FBS and 1% antibiotic antimycotic solution. Passaging of cells were done using 1X TPVG at about 80% confluency.

### 2.11 Treatment with palmitic acid conjugated BSA and CORM-A1

Palmitate stock solution was prepared as described previously [28]. Briefly, 100 mM PA was conjugated with 10% BSA to obtain PA (10 mM FFA/1% BSA) stock solution. PA was further diluted with media to obtain 100 μM working solution and the same was used for treatment of HepG2 cells.

### 2.12 Cytotoxicity assessment

HepG2 cells were seeded in 96 well plate (10^4^ cells/well) in DMEM. PA (100 μM) alone or in combination with CORM-A1 (100 μM) were dosed. After 24 h 3-(4,5-dimethylthiazol-2-yl)-2,5-diphenyltetrazolium bromide (MTT; 5 mg/ml) was added and cells were incubated in the dark for 4 h. Resultant formazan crystals were dissolved in DMSO (150μL/well) and absorbance was measured at 540 nm (Synergy HTX Multimode Reader) [29].

### 2.13 Intracellular ROS generation by CellROX, DHE and MitoSOX staining

HepG2 cells were treated as per same treatment schedule and at the end, cells were incubated with CellROX (5 μM) for 30 min/DHE (10 μM) for 10 min/MitoSOX (5 μM) for 30 min. At the end of the incubation, the cells were washed with PBS, mounted using fluoroshield mounting medium with DAPI and photographed (Zeiss Axioplan-2 imaging fluorescence microscope).

### 2.14 Fluorescence activated cell-sorting (FACS) analysis

At the end of the treatment period, HepG2 cells were stained with 5 μM MitoSOX, or 50 nM MitoTracker for 30 min at 37 °C (protected from light). After washing twice, the samples were analysed using a flow cytometer (FACScalibur, BD Biosciences).

### 2.15 Mitochondrial membrane potential assessment by JC-1 and TMRE staining

Cells were seeded in 6 wells plate and treated as mentioned earlier. Control and treated cells were washed with 1X PBS and incubated with JC-1 (5 μg/ml) in pre‐warmed 1X PBS for 30 min at 37 °C. Cells were photographed using Evos FLoid cell imaging station and fluorescent intensity was quantified using ImageJ software. TMRE (100 nM) staining was done in 1X PBS for 30 min at 37 °C. At the end of the incubation, cells were trypsinized, centrifuged at 1000g for 5 min and re-suspended in pre‐warmed PBS. Fluorescence was recorded using a flow cytometer (FACScalibur, BD Biosciences).

### 2.16 Mitochondrial respiration (seahorse XF analyzer)

The Seahorse XFp Analyzer (Seahorse Biosciences, North Billerica, MA) was used according to the manufacturer's protocol to measure OCR and ECAR of the cells. Briefly, cells were seeded in Seahorse Flux Analyzer mini plates (10000 cells/well) and incubated overnight at 37 °C. Later, cells were treated according to the specific *in vitro* protocols, as mentioned earlier. Thereafter, the culture medium was changed to XFp base medium minimal DMEM (Seahorse Biosciences) and placed in a non-CO2 incubator at 37 °C. Mitochondrial function was assessed using the Seahorse XFp Analyzer by monitoring changes in OCR and ECAR as previously described. Three OCR measurements were obtained under basal conditions and upon sequential injection of 2 μM oligomycin, 2 μM fluoro-carbonyl-cyanide phenylhydrazone (FCCP) and 0.5 μM rotenone plus 0.5 μM antimycin A. OCR values were calculated from 3 min measurement cycles. The OCR measurements were adjusted to cell numbers. Glycolysis was assessed by analyzing ECAR in hepatocytes cultured in glucose-free medium after sequential addition of 10 mM glucose, 2 μM oligomycin and 100 mM 2-deoxyglucose. The final data were obtained using the Seahorse XFp software and calculated according their instructions.

### 2.17 Statistical analysis

The data were expressed as mean ± SEM and analysed by one-way analysis of variance (ANOVA), followed by Bonferroni's multiple comparison test using Graph Pad Prism 5.0 (CA, USA). *P < 0.05, **P < 0.01 and ***P < 0.001 were considered to be significant.

## 3 Results

### 3.1 CORM-A1 treatment improves NASH associated pathological changes in HFHF fed mice

**Fig. 1:**
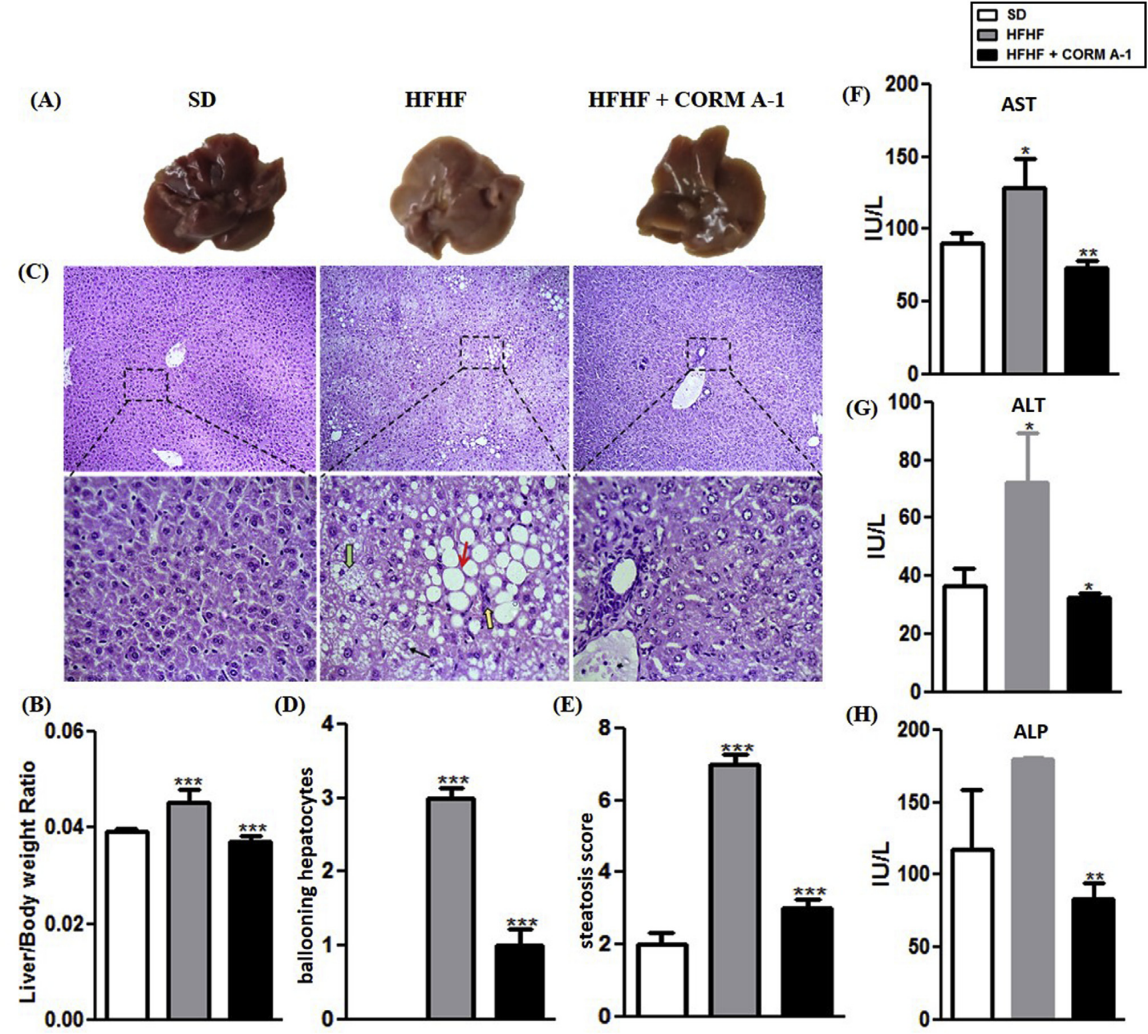
**Mice treated with CORM-A1 showed improvement in histopathology and functional status of liver.** (A) Autopsy (B) Liver/Body weight ratio (C) H&E staining of liver section of C57BL/6J mice fed with SD, HFHF diet or HFHF + CORM-A1 (magnification 100X and 400X), **Black arrow** indicate Mallory hyaline, **Green arrow** indicate ballooning hepatocytes, **yellow arrow** indicates inflammation and **red arrow** indicate steatosis in liver (D) Scoring of ballooning hepatocytes (E) Scoring of steatosis and liver function enzymes (F) AST (G) ALT and (H) ALP. Results expressed as mean ± S.E.M. *p < 0.05, **p < 0.01 or ***p < 0.001 is when HFHF compared to SD and HFHF + CORM-A1 is compared to HFHF group. (For interpretation of the references to colour in this figure legend, the reader is referred to the Web version of this article.)

**Supplementary Fig. 1:**
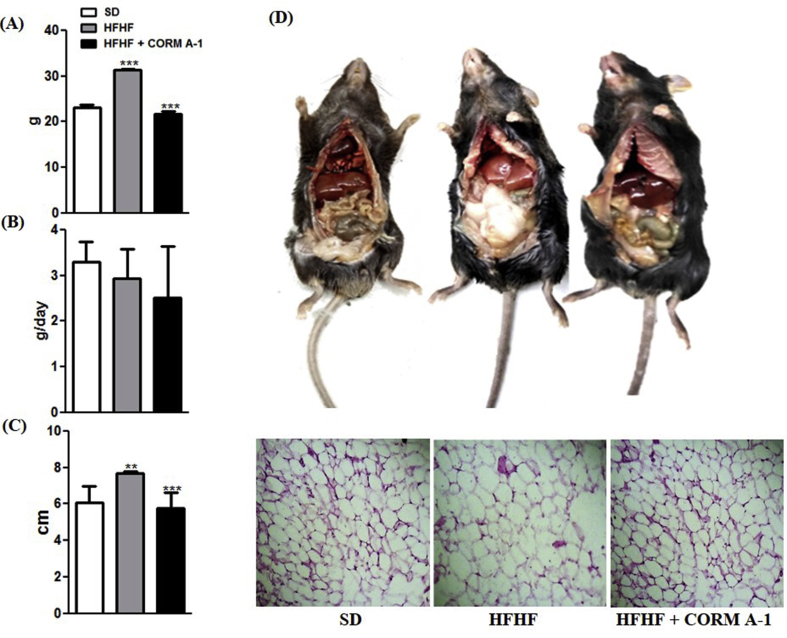
**CORM-A1 treatment attenuates weight gain in HFHF fed mice.** Male C57BL/6J mice (n = 6/group) fed with SD, HFHF diet or HFHF + CORM-A1 (2 mg/kg; ip, from 9th to 16 weeks). (A) Body weight gain at the end of 16 weeks experiment (B) Food intake (C) Abdominal circumference and (D) C57BL/6J mice viscera showing adipose build-up and cross section area of respective adipocytes. (magnification 200X). Results expressed as mean ± S.E.M. *p < 0.05, **p < 0.01 or ***p < 0.001 is when HFHF compared to SD and HFHF + CORM-A1 is compared to HFHF group.

**Supplementary Fig. 2:**
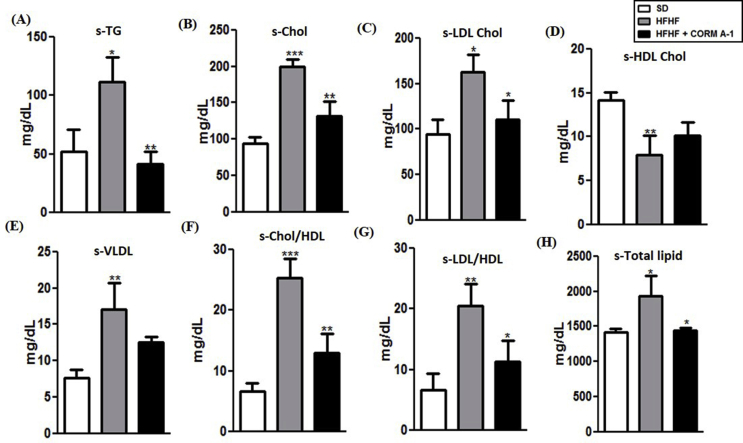
**Mice treated with CORM-A1 maintained a healthier metabolic profile on HFHF diet.** C57BL/6J mice (n = 6/group) fed with SD, HFHF diet or HFHF + CORM-A1 (2 mg/kg; ip, from 9th to 16 weeks). At the end of 16 weeks blood was collected and serum lipid profiles were done. (A) s-TG (B) s-Chol (C) s-LDL Chol (D) s-HDL Chol (E) s-VLDL (F) s-Chol/HDL (G) s-LDL/HDL and (H) s-Total lipids. Results expressed as mean ± S.E.M. *p < 0.05, **p < 0.01 or ***p < 0.001 is when HFHF compared to SD and HFHF + CORM-A1 is compared to HFHF group.

**Supplementary Fig. 3:**
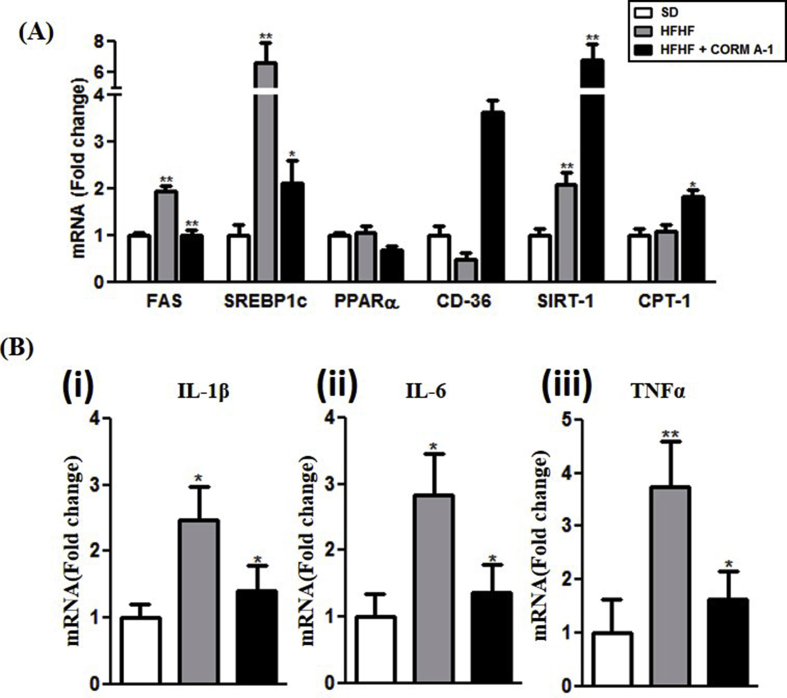
**CORM A-1 improves lipid metabolism and inflammation in HFHF fed mice.** C57BL/6J mice (n = 6/group) fed with SD, HFHF diet or HFHF + CORM-A1 (2 mg/kg; ip, from 9th to 16 weeks). (A) changes in mRNA expression of genes governing hepatic lipid metabolism in liver and (B) mRNA expression of inflammatory genes (i) IL-1β (ii) IL-6 (iii) TNF-α. Results expressed as mean ± S.E.M. *p < 0.05, **p < 0.01 or ***p < 0.001 is when HFHF compared to SD and HFHF + CORM-A1 is compared to HFHF group.

CORM-A1 treatment to HFHF fed mice resulted in reduced weight gain and abdominal circumference (P < 0.0001) and showed less fat accumulation as compared to HFHF fed mice. However, no difference in food intake was observed for either of the groups (Figs. S1A–C). Results obtained in iCORM A-1 treated group were comparable to that of HFHF fed group (data not shown), thus confirming that the inactivated form was not able to elicit any response. H&E staining of visceral adipose tissue revealed larger adipocyte diameter in HFHF fed mice, but treatment with CORM-A1 significantly reduced the same and showed a mixed population of larger and medium sized adipocytes (Fig. S1D). Next, we evaluated the influence of CORM-A1 on indices of metabolic profile. CORM-A1 treatment significantly improved HFHF diet-induced dysregulated serum lipid profile (Figs. S2A–H). Liver of HFHF fed mice was pale-yellow coloured and had a higher liver to body weight ratio (~35%) as compared to SD mice but the same morphometric indices in CORM-A1treated mice were comparable to SD mice (Fig. 1 A&B). Microscopic evaluation of liver tissue revealed significantly higher amount of micro vesicular hepatic steatosis, ballooning hepatocytes and mallory hyaline in HFHF fed mice. Whereas, no substantial evidence of hepatic steatosis was seen in CORM-A1 treated mice (Fig. 1C). Scoring of liver sections revealed significantly higher number of ballooning hepatocytes and increased steatosis in HFHF fed mice that was significantly decreased (P < 0.0001) in CORM-A1 treated group (Fig. 1 D&E). Moreover, these structural changes were associated with circulating lower levels of liver injury marker enzymes (AST, ALT and ALP) (Fig. 1F–H). Next, we investigated the impact of CORM-A1 on expression of genes associated with lipid metabolism and inflammation. CORM-A1 treatment significantly decreased the mRNA expression of key lipogenic genes (FAS and Srebp-1C) compared to HFHF fed mice. Further, mRNA expression of CD36 and CPT-1 showed non-significant changes whereas, SIRT1 showed significant upregulation in HFHF fed mice. But, CORM-A1 treatment accounted for significant increase in the mRNA levels of CD36, SIRT1 and CPT-1 in HFHF fed mice (P < 0.01) (Fig. S3A). Additionally, CORM-A1 treatment resulted in significant (P < 0.009) decrement in mRNA expression of pro-inflammatory genes, such as: interleukin 1-beta (IL-1β), interleukin-6 (IL-6) and tumor necrosis factor-alpha (TNF-α) compared to HFHF fed mice (Fig. S3B). Overall, these results show CORM-A1 improved lipid metabolism, hepatic steatosis and associated inflammation in HFHF fed mice.

### 3.2 CORM-A1 facilitates Nrf2 translocation, modulates Keap1 expression and activates ARE genes in liver of HFHF fed mice

**Fig. 2:**
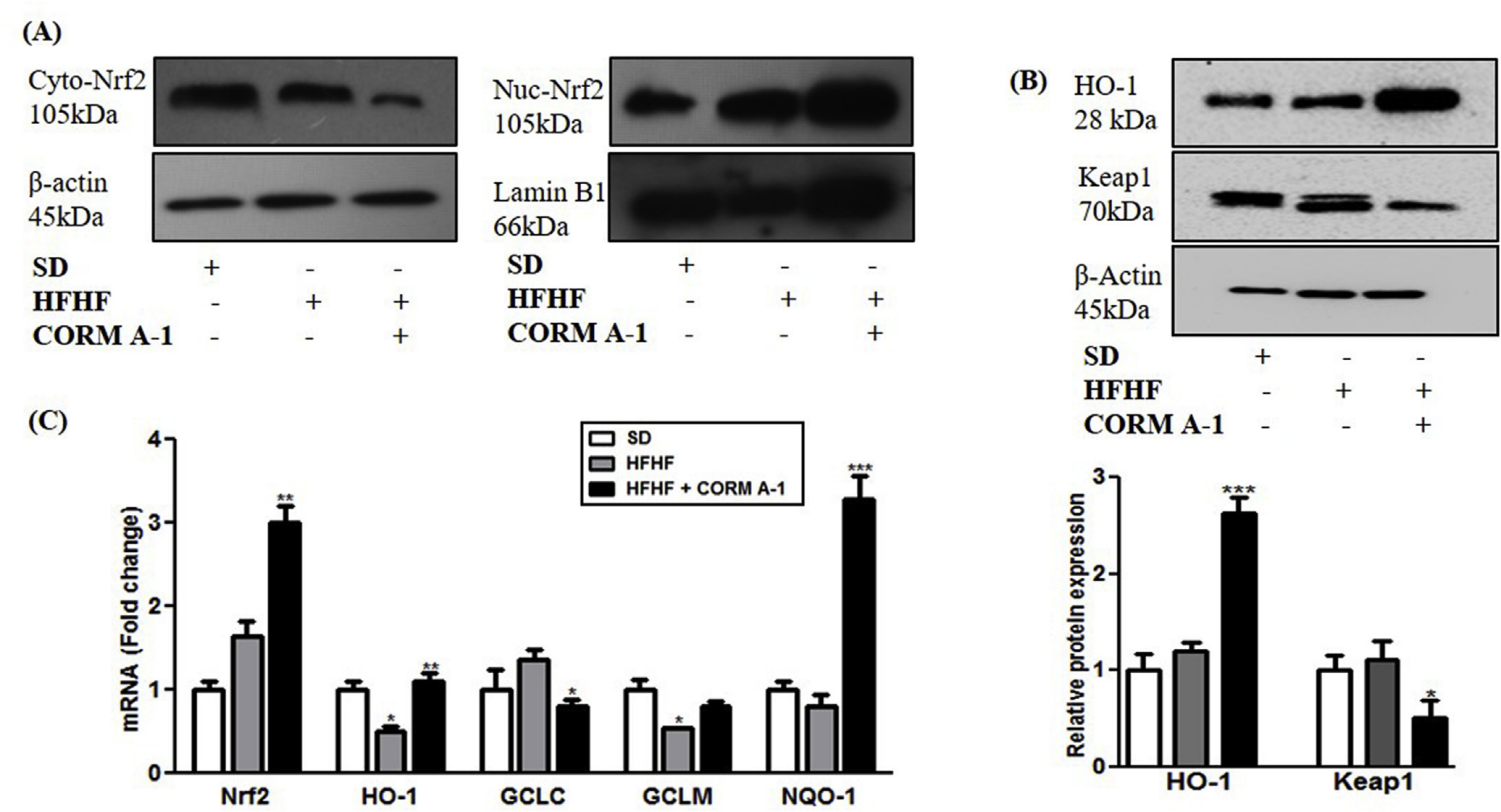
**CORM-A1 promotes Nrf2 translocation by modulating Keap1 expression and regulate ARE genes in liver of HFHF fed mice.** C57BL/6J mice (n = 4/group) fed with SD, HFHF diet or HFHF + CORM-A1 (2 mg/kg; ip, from 9th to 16 weeks). (A) Immunoblot images of cytoplasmic and nuclear Nrf2 proteins (B) HO-1 and Keap1 proteins and their quantifications and (C) The mRNA expression of Nrf2 and related ARE genes. Results expressed as mean ± S.E.M.*p < 0.05, **p < 0.01 or ***p < 0.001 is when HFHF compared to SD and HFHF + CORM-A1 is compared to HFHF group.

Previous studies had shown that CORM-A1 exerts antioxidant activity by facilitating Nrf2 activation [25]. We evaluated Nrf2 expression in cytosolic and nuclear fraction of livers from SD, HFHF fed and HFHF + CORM-A1 treated mice. Liver of CORM-A1 treated mice showed lowered levels of Nrf2 protein in cytosol and significantly higher levels in nucleus as compared to HFHF fed mice (Fig. 2A). mRNA expression and protein content of HO-1 showed non-significant changes in HFHF fed mice, however CORM-A1 treatment significantly stimulated its expression (P < 0.0062). Conversely, Keap1 protein was significantly reduced in CORM-A1 treated group (P < 0.0113) (Fig. 2B) and this effect was associated with increased mRNA expression of ARE genes, i.e. GCLM and NQO-1 in CORM-A1 treated mice (Fig. 2C). These set of findings demonstrate that CORM-A1 promoted Nrf2 activation and subsequent upregulation of antioxidant genes in HFHF fed mice.

### 3.3 CORM-A1 induce changes in mRNAs associated with hepatic mitochondrial biogenesis and function *in vivo*

**Fig. 3:**
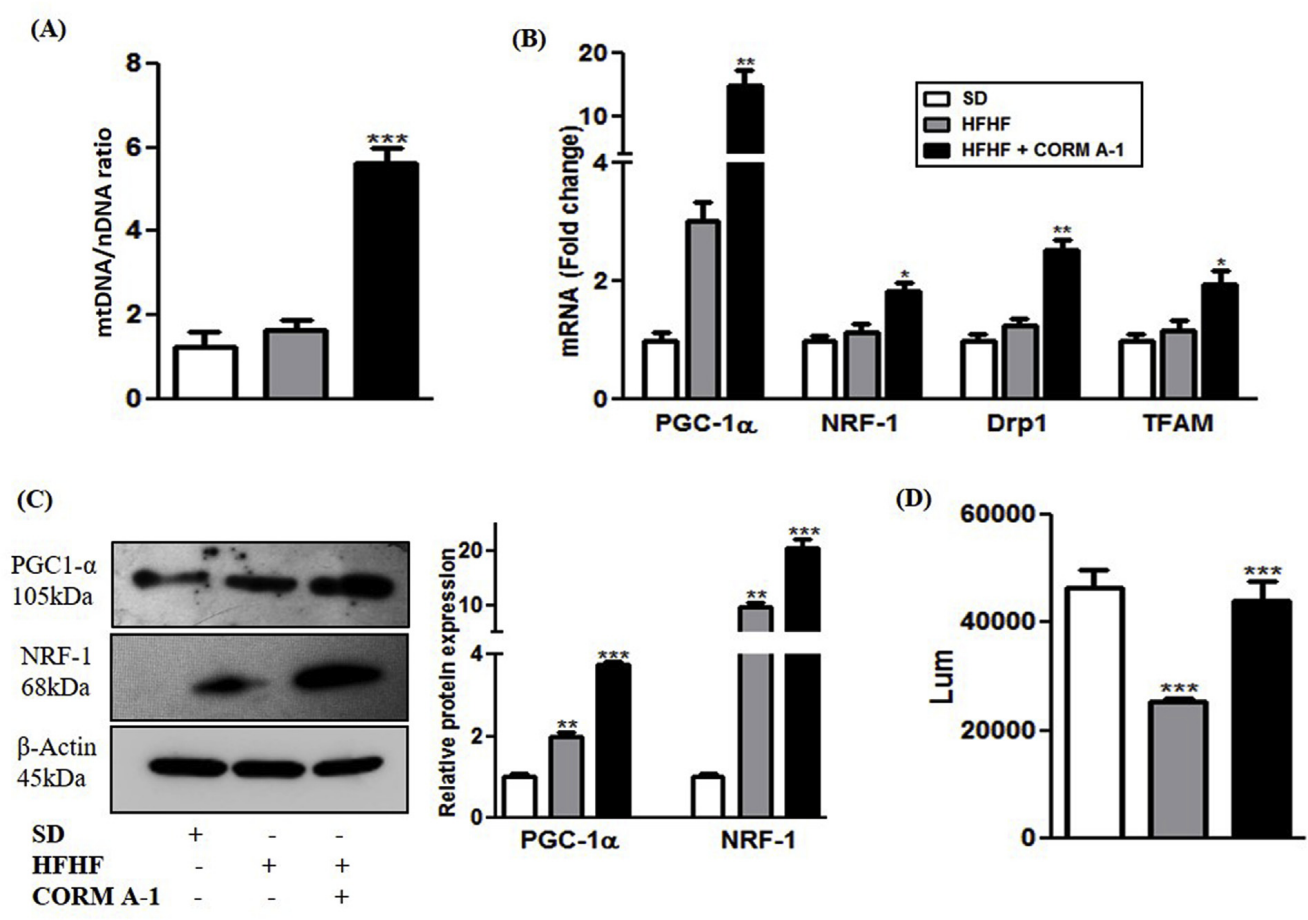
**CORM-A1 treatment improves hepatic mitochondrial biogenesis and function.** C57BL/6J mice (n = 4/group) fed with SD, HFHF diet or HFHF + CORM-A1 (2 mg/kg; ip, from 9th to 16 weeks). (A) mtDNA content (B) Hepatic mRNA expression of genes related to mitochondrial biogenesis (C) Immunoblot images of regulatory proteins of mitochondrial biogenesis and their quantifications and (D) cellular ATP levels. Results expressed as mean ± S.E.M. *p < 0.05, **p < 0.01 or ***p < 0.001 is when HFHF compared to SD and HFHF + CORM-A1 is compared to HFHF group.

The effects of CORM-A1 on mitochondrial biogenesis and energetics was evaluated in liver of steatotic or CORM-A1 treated mice. mtDNA copy number in liver of HFHF fed mice was comparable to SD mice but, CORM-A1 treatment accounted for a significant increment (P < 0.0001) compared to HFHF fed mice (Fig. 3A). PGC-1α and NRF-1 mRNA expression directly regulate the mitochondrial number as well as other metabolic events in steatotic mice [30]. We also observed a significant increment in mRNA expression and protein content of PGC-1α and NRF-1 in HFHF fed mice, which were further significantly elevated in CORM-A1 treated mice as compared to HFHF fed mice. mRNA levels of regulators of mitochondrial fission (Drp1) and mtDNA (TFAM) was unchanged and elevated in the liver of HFHF fed and CORM-A1 treated mice respectively (Fig. 3B&C). Additionally, the functional status of mitochondria was studied by assessing hepatic ATP production. Data showed that ATP content in liver of HFHF fed mice was significantly lower than SD mice. CORM-A1 treatment was instrumental in increasing the ATP production significantly (P < 0.002) to match the levels measured in SD mice (Fig. 3D). These findings indicate that HFHF fed mice showed unaltered mitochondrial number but compromised ATP production and this was significantly improved with CORM-A1 treatment.

### 3.4 CORM-A1 improves oxidative stress and cell survival via Nrf2 translocation and activation of ARE genes in hepatocytes

**Fig. 4:**
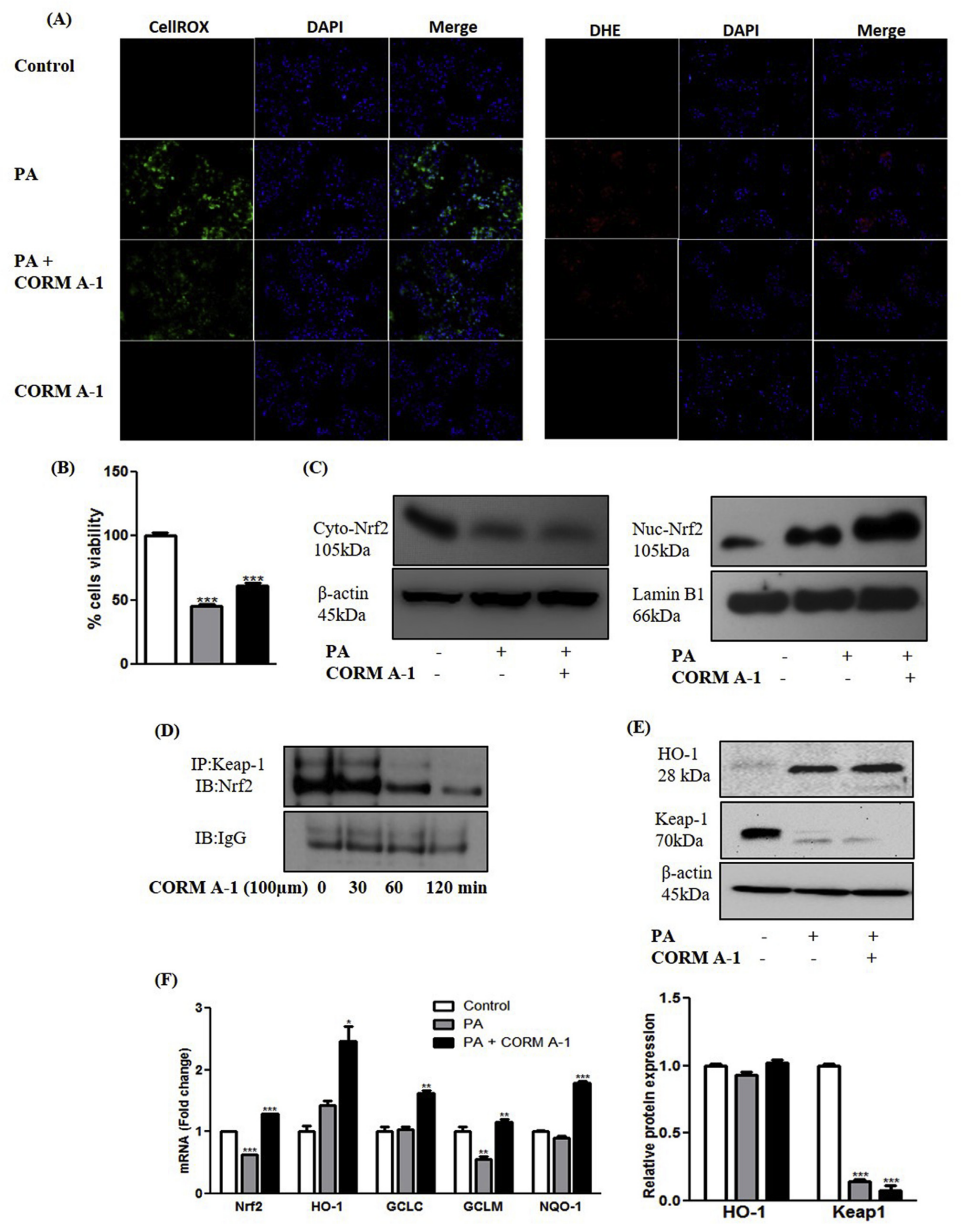
**CORM-A1 improves oxidative stress and cell survival via Nrf2 translocation and activation of ARE genes in hepatocytes.** HepG2 cells were treated with PA (100 μm) in presence and absence of CORM-A1 (100 μm). Oxidative stress in HepG2 cells was evaluated using (A) CellROX and DHE stain in control, PA, PA + CORM-A1 and CORM-A1 groups. Cell survival was determine using (B) cell viability assay (MTT) (C) Nrf2 translocation was checked using immunoblot analysis in cytoplasm and nucleus (D) co-immunoprecipitation demonstrating time depended decrement in keap1 protein (E) qualitative and quantitative levels of HO-1 and Keap1 proteins and (F) expression levels of antioxidant gene was recorded using qPCR analysis. Results expressed as mean ± S.E.M. *p < 0.05, **p < 0.01 or ***p < 0.001 is when PA treatment is compared to control cells and PA + CORM-A1 is compared to PA alone group.

Our findings are suggesting that the anti-NASH potential of CORM-A1 in HFHF fed mice involves the activation of Nrf2-ARE pathway. Progression of hepatic steatosis comprises of multiple overlapping molecular events. Hence, we carried out molecular studies using human hepatocellular cell line (HepG2) to confirm and explain our *in vivo* findings. PA-treatment to hepatic cells mimics the condition of second hit of NALFD progression via higher ROS generation and cell death [31]. PA treated HepG2 cells resulted in ~50% reduction in cell viability but CORM-A1 (100 μm) treated group recorded relatively more (~68%) viable cells. PA treatment resulted in heightened levels of intracellular ROS as evidenced by DHE (red) and CellROX (green) staining but CORM-A1 co-supplementation resulted in significant decrement in red and green fluorescence respectively (Fig. 4A&B), thus suggesting decreased ROS production. Translocation of Nrf2 protein from cytoplasm to nucleus was further confirmed in HepG2 cells wherein, CORM-A1 treatment accounted for significantly higher nuclear accumulation of Nrf2 as compared to PA treated cells (Fig. 4C). Western blot analysis of Keap1 protein (negative regulator of Nrf2) showed a significant decrement in PA and CORM-A1 treated groups. Additionally, co-immunoprecipitation (co-IP) studies results revealed a time-depended decrement in Keap1/Nrf2 interaction after treatment with CORM-A1 to HepG2 cells (Fig. 4D). Further, an increase in HO-1 mRNA and protein were recorded in PA treated cells whereas, more pronounced effect in mRNA and an increment in protein was noted in CORM-A1 co-supplemented group. Other ARE genes viz. GCLC, GCLM and NQO-1 were also studied wherein, GCLM mRNA levels was decreased significantly following PA treatment. But, GCLC and NQO-1 mRNA levels in the same group showed non-significant changes. CORM-A1 co-supplementation to PA treated HepG2 cells was marked by significantly elevated mRNA levels of GCLC, GCLM and NQO-1 (Fig. 4E&F).

### 3.5 PA-mediated impaired mitochondrial oxidative stress, membrane potential and mass in HepG2 cells is alleviated by the antioxidant potential of CORM-A1

**Fig. 5:**
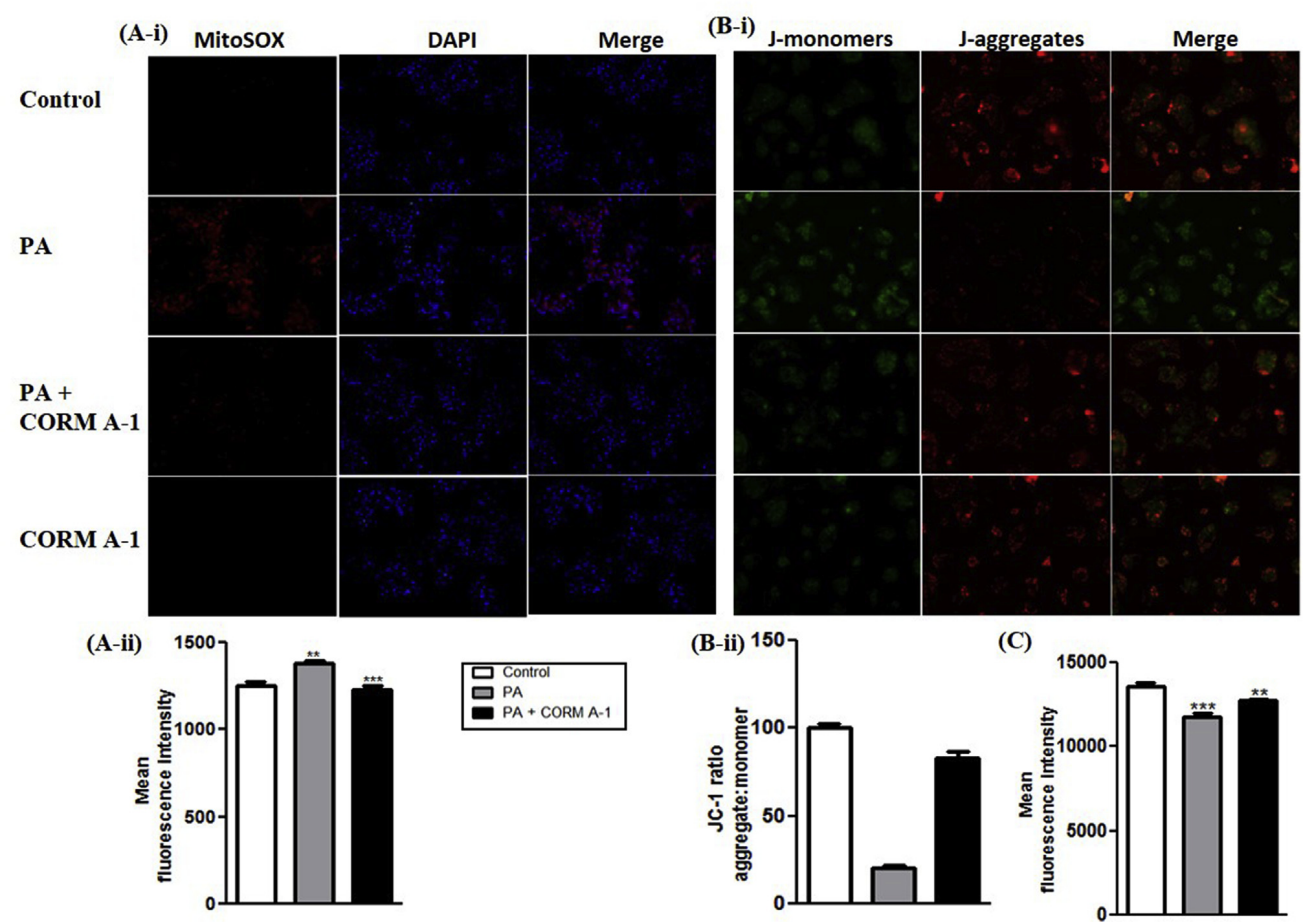
**CORM-A1 improves mitochondrial oxidative stress and membrane potential in PA treated HepG2 cells.** Mitochondria specific ROS and MMP was measured in HepG2 cells treated with PA (100 μm) in presence and absence of CORM-A1 (100 μm) for 12 h. Cells stained with (A i&ii) MitoSOX and their fluorescence intensity (B i&ii) JC-1 staining for measuring MMP and its fluorescence intensities measured using FACS analysis and (C) fluorescence intensities of TMRE stain. Results expressed as mean ± S.E.M. *p < 0.05, **p < 0.01 or ***p < 0.001 is when PA treatment is compared to control cells and PA + CORM-A1 is compared to PA alone group.

**Fig. 6:**
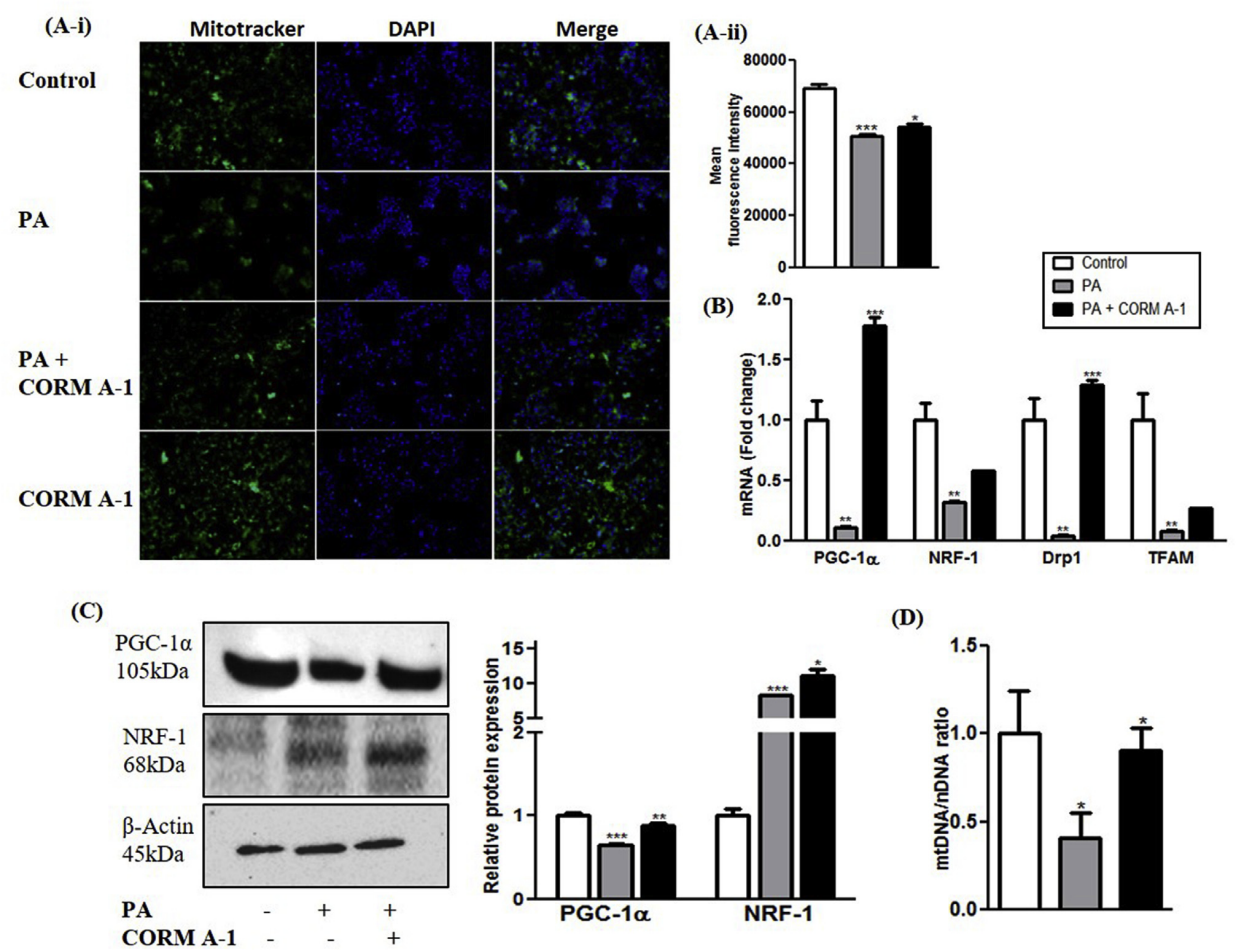
**CORM-A1 improves mitochondrial mass and biogenesis in PA treated HepG2 cells.** Cells were treated for 24 h with PA and PA + CORM-A1 wherein, (A i&ii) Mitotracker staining and its fluorescence intensity (B) mRNA expression of regulatory genes for mitochondrial biogenesis (C) immunoblot images and their quantification and (D) mtDNA content. Results expressed as mean ± S.E.M. *p < 0.05, **p < 0.01 or ***p < 0.001 is when PA treatment is compared to control cells and PA + CORM-A1 is compared to PA alone group.

PA-treated HepG2 cells are a known model to study mitochondrial dysfunction [32]. Mitochondrial specific ROS was accessed by MitoSOX staining of HepG2 cells. Imaging and FACS analysis revealed prominent red fluorescence (higher ROS) in PA treated HepG2 cells whereas a weaker fluorescence (lower ROS) was recorded in CORM-A1 co-supplemented group (Fig. 5A-i&ii). JC-1 staining forms red coloured J-aggregates whereas, a decrease in red/green fluorescence intensity ratio marks mitochondrial depolarization. We recorded a shift in fluorescence intensity ratio (towards green) in PA treated HepG2 cells as compared to the control. CORM-A1 co-supplementation for 12 h accounted for higher indices of fluorescence (red/green ratio) suggesting improved mitochondrial membrane potential (MMP) (Fig. 5B-i&ii). Likewise, FACS analysis using TMRE stain was performed to assess ΔΨm wherein, PA-treated HepG2 cells recorded a significant decrement (P < 0.0001) compared to control cells. CORM-A1 co-treatment improved ΔΨm as evidenced by significant increment in fluorescence intensity of HepG2 cells (Fig. 5C). Mitotracker dye stains mitochondria irrespective of MMP and hence, the fluorescence intensity is indicative of the total mitochondrial mass. Weak fluorescence (P < 0.0001) recorded in PA-treated HepG2 cells in contrast was prominent in CORM-A1 treated cells (Fig. 6A-i&ii).

### 3.6 CORM-A1 improves PA-abrogated mitochondrial biogenesis and respiration in HepG2 cells

**Fig. 7:**
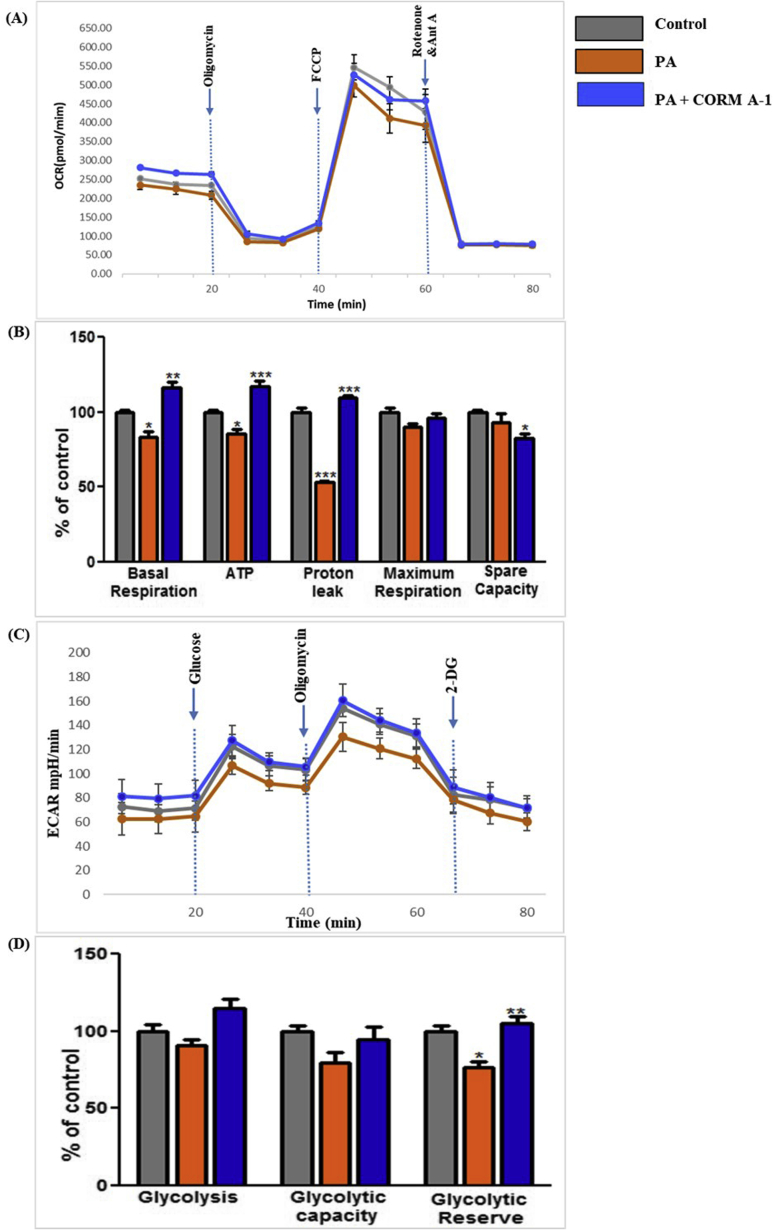
**CORM-A1 improves PA-abrogated respiration in HepG2 cells.** HepG2 cells were treated with PA (100 μm) in presence and absence of CORM-A1 (100 μm) for 24 h. Cell respiration were measured as Oxygen consumption rate (OCR) and extracellular acidification rate (ECAR) and quantification was done using Seahorse XFe 96 Metabolic Flux Analyzer. (A) mitochondrial respiration measured as OCR (B) quantification of other respiratory parameters (Basal respiration, ATP, Proton leak, maximum respiration and spare capacity) using OCR data. (C) glycolytic lactic acid production (non-mitochondrial respiration) measured as ECAR (D) calculated values for glycolysis, glycolytic capacity and reserves from ECAR data. Results expressed as mean ± S.E.M. *p < 0.05, **p < 0.01 or ***p < 0.001 is when PA treatment is compared to control cells and PA + CORM-A1 is compared to PA alone group.

Mitochondrial biogenesis regulatory mRNAs and protein contents in HepG2 cells were also assessed in response to the different treatments. PA significantly reduced mRNA levels of PGC-1α and NRF-1, however CORM-A1 co-supplementation rescued their expression (Fig. 6B). Protein levels of PGC-1α was decreased and NRF-1 was increased in PA treated HepG2 cells. CORM A1 treatment accounted for a significant increase (P < 0.01) in protein levels of PGC-1α and NRF-1 (Fig. 6C). mRNA levels of other key genes controlling mitochondrial fission and mtDNA copy number, such as Drp1 and TFAM were significantly downregulated in PA-treated HepG2 cells. Though, CORM-A1 did not induce major changes in mRNA expression of TFAM but a significant increment was observed in Drp1 (P < 0.001). Furthermore, in PA treated HepG2 cells we observed a significant decrement in mtDNA content that was significantly improved (P < 0.05) by CORM-A1 treatment (Fig. 6D). These findings are in agreement with our results obtained *in vivo*. As mitochondrial oxidative stress and redox imbalance are implicated in progression to NASH [33], we further assessed mitochondrial function by seahorse XF extracellular flux analyzer, as measures of oxygen consumption rate (OCR) and glycolytic activity assessing lactic acid production and extracellular release (ECAR). CORM-A1 accounted for higher basal respiration rate than PA treated cells or control group. In the next step, addition of oligomycin resulted in respiration linked to ATP production and proton leak. A significant decrement in ATP production was also observed by PA treatment but CORM-A1 accounted for a significant increment (P < 0.001). Further, PA treatment showed diminished proton leak, but CORM-A1 treated cells showed a significant increment (P < 0.001). Addition of FCCP is known to maximize mitochondrial respiratory capacity whereas; rotenone blocks complex I and inhibits oxidative phosphorylation [34]. The same also enables the detection of spare respiratory capacity. In our study, PA treatment resulted in significant decrement in indices of maximal cellular respiration and spare respiratory capacity, but CORM-A1 treatment significantly improved these parameters in PA treated HepG2 cells (Fig. 7A&B).

The extracellular acidification rate is indicative of glycolytic activity in absence of mitochondrial respiration. Maximum ECAR or glycolytic capacity observed in PA treated cells was significantly diminished, in agreement with indices of glycolysis and glycolytic reserve. CORM-A1 treatment improved the glycolytic capacity and glycolytic reserve, however maximum ECAR values showed a non-significant increment. Indices of non-glycolytic acidification in PA-treated cells with or without CORM-A1 showed a non-significant increment (Fig. 7C&D). These results suggest that co-supplementation of CORM-A1 increases mitochondrial oxidative phosphorylation in PA treated HepG2 cells.

## 4 Discussion

We have investigated the protective effects of the antioxidant gasotransmitter CORM-A1 in a mouse model of NASH. Owing to the multifactorial nature of NASH and resultant morbidity and mortality [35], an effective therapeutic strategy should involve a multipronged approach with a synergistic effect on various facets of liver function. Herein, we show that treatments of HFHF mice, an established model of NASH, with CORM-A1 resulted in significant hepatoprotection at multiple levels.

In our study, HFHF diet was fed to C57BL/6J mice to mimic fructose and fat rich foods that are known to be associated with life style disorders [36]. In these mice, we observed significant elevation in titers of circulating liver injury markers along with fatty manifestations in liver histology thus, providing evidence on steatotic changes. In addition, steatotic mice showed elevated serum lipid profile, ballooned hepatocytes, impaired redox and lipid homeostasis and lowered ATP production. These observations are in agreement with other reports on HFHF diet-induced NASH where fructose feeding is known to deplete ATP production and suppress mitochondrial fatty acid oxidation resulting in increased production of ROS [37]. Remarkably, CORM-A1 treatment to HFHF fed mice showed a significant improvement in the said histopathological and biochemical/functional parameters.

In acetaminophen-treated mice, we have previously reported that, the hepatoprotective effects of CORM-A1 involve its ability to promote antioxidant responses through the activation of Nrf2 [25]. Experimental and clinical studies have reported a strong association between severity of NASH and degree of oxidative stress [38]. Herein, CORM-A1-mediated induction of Nrf2-ARE pathway provides protection against oxidative stress induced by PA/HFHF diet. Augmented nuclear translocation/activation of Nrf2 was observed in *in vivo* and *in vitro* CORM-A1treated groups and was confirmed by consequent up-regulation of ARE genes such as: HO-1, GCLC, GCLM and NQO-1. Further, CORM-A1-mediated Nrf2 nuclear translocation was seen in HepG2 cells with time-dependent dissociation from its negative regulator Keap1, thus, confirming our previous study using *in silico* docking analysis of CO and showing that CORM-A1 promotes Nrf2 activation via CO-mediated release of Nrf2 from Keap1 [25].

Oxidation of fatty acids is a high energy yielding process along with production of excessive ROS that is kept under control by the cellular antioxidant defense system. However, systemic and intracellular lipid overload, ROS and redox imbalance leads to GSH depletion, apoptosis and cell death [39]. Additionally, previous studies had reported that various Nrf2 activators can improve lipid metabolism [40]. In our study we observed significant decrease in fatty acid synthesis and enhanced clearance/catabolism of lipids from the steatotic liver following CORM-A1 treatment.

Apart from its role as a transcriptional activator of cellular antioxidants, Nrf2 is also involved in maintaining mitochondrial homeostasis [41]. In NASH, mitochondrial dysfunction plays a crucial role because of its involvement in β-oxidation of fatty acids and the same has also been implicated in first and second hits of NASH [42]. Hence, we evaluated the influence of CORM-A1-induced Nrf2 signaling on mitochondrial functions in PA treated HepG2 cells. PA has been preferred over stearic and oleic acids for its ability to induced mitochondrial dysfunction in HepG2 cells [32]. CORM-A1 co-supplemented HepG2 cells showed reduced intracellular and mitochondria ROS formation along with a significant improvement in MMP. Since MMP has been implicated as an indicator of cellular metabolic status, the reduced ROS (DHE and CellROX) and improved MMP (JC-1 and TMRE) can be attributed to Nrf2-mediated control of cellular redox homeostasis [42].

Additionally, CORM-A1 increased mtDNA copy number in PA-treated HepG2 cells or in liver of HFHF fed mice, a mechanism also attributable to Nrf2 activation and resulting in mitochondrial biogenesis, as suggested by the reported effects of Nrf2-dependent translational activation of NRF-1 [43] and by the elevation of the transcriptional co-activator PGC-1α [30]. The specific contribution of Nrf2 in maintaining NRF-1 and PGC-1α expressions was reported in liver of Nrf2 knockout mice [41]. Our data also showed that CORM-A1 treatment accounted for significantly higher levels of TFAM and Drp1 mRNAs. TFAM is a mitochondria specific transcription factor involved in replication of mitochondrial genome and in maintenance of mtDNA copy number [44], whereas Drp1 regulates mitochondrial fission and is a potential therapeutic target in metabolic diseases [45].

PA has been reported to cause ROS production and hyperpolarization of mitochondria. This scenario is mitigated at cellular level by increasing proton leak [46,47]. Also, PA causes accumulation of pyruvate leading to a decrease in basal and maximal mitochondrial respiratory rate that shifts the OCR to ECAR by increasing glycolytic flux [48]. Our findings of improved basal respiration in PA + CORM-A1 treated HepG2 cells, accompanied by a significant increment in proton leak is the cause for observed higher ATP production. CO induced increase in proton leak is the reported cause for a higher uncoupling activity in isolated mitochondria and in experimentally induced insulin resistance in obese mice [[49], [50], [51]]. PA depressed ATP levels and abrogated mitochondrial respiration due to a compensatory higher metabolic flux in hepatocytes that led to poor cell viability. An increment in non-mitochondrial ATP generation through glycolysis in early phase has been reported. In advanced stages of hepatic steatosis the ATP reserves are depleted [52]. However, CORM-A1 co-supplementation to PA treated HepG2 cells accounted for higher indices of glycolytic reserves thus implying towards a healthy state of mitochondria and less dependency on non-mitochondrial respiration.

In summary, the results of our study evidencing CORM-A1-mediated amelioration of mitochondrial function in steatotic liver via Nrf2, is the first to directly suggest the therapeutic potential of CORM-A1 for the clinical management of NASH and fatty liver.

## Declaration of interests

The authors declare no competing interests.

## References

[1] Cheng Y., Zhang K., Chen Y., Li Y., Li Y., Fu K., Feng R. (2016). Associations between dietary nutrient intakes and hepatic lipid contents in NAFLD patients quantified by 1H-MRS and dual-Echo MRI. Nutrients.

[2] Day C.P. (2010). Genetic and environmental susceptibility to non-alcoholic fatty liver disease. Dig. Dis..

[3] Younossi Z., Anstee Q.M., Marietti M., Hardy T., Henry L., Eslam M., George J., Bugianesi E. (2018). Global burden of NAFLD and NASH: trends, predictions, risk factors and prevention. Nat. Rev. Gastroenterol. Hepatol..

[4] Fuchs M., Sanyal A.J. (2012). Lipotoxicity in NASH. J. Hepatol..

[5] Oseini A.M., Sanyal A.J. (2017). Therapies in non‐alcoholic steatohepatitis (NASH). Liver Int..

[6] Pessayre D., Fromenty B. (2005). NASH: a mitochondrial disease. J. Hepatol..

[7] Begriche K., Igoudjil A., Pessayre D., Fromenty B. (2006). Mitochondrial dysfunction in NASH: causes, consequences and possible means to prevent it. Mitochondrion.

[8] Diehl A.M. (2000). Cytokine regulation of liver injury and repair. Immunol. Rev..

[9] Chambel S.S., Santos-Gonçalves A., Duarte T.L. (2015). The dual role of Nrf2 in nonalcoholic fatty liver disease: regulation of antioxidant defenses and hepatic lipid metabolism. BioMed Res. Int..

[10] de Haan J.B. (2011). Nrf2 activators as attractive therapeutics for diabetic nephropathy. Diabetes.

[11] Jadeja R.N., Upadhyay K.K., Devkar R.V., Khurana S. (2016). Naturally occurring Nrf2 activators: potential in treatment of liver injury. Oxid. Med. Cell. Longev..

[12] Liu W., Wang D., Liu K., Sun X. (2012). Nrf2 as a converging node for cellular signaling pathways of gasotransmitters. Med. Hypotheses.

[13] Tenhunen R., Marver H.S., Schmid R. (1968). The enzymatic conversion of heme to bilirubin by microsomal heme oxygenase. Proc. Natl. Acad. Sci..

[14] Boczkowski J., Poderoso J.J., Motterlini R. (2006). CO–metal interaction: vital signaling from a lethal gas. Trends Biochem. Sci..

[15] Owens E.O. (2010). Endogenous carbon monoxide production in disease. Clin. Biochem..

[16] Ryter S.W., Choi A.M. (2013). Carbon monoxide in exhaled breath testing and therapeutics. J. Breath Res..

[17] Motterlini R., Otterbein L.E. (2010). The therapeutic potential of carbon monoxide. Nat. Rev. Drug Discov..

[18] Taguchi K., Nagao S., Maeda H., Yanagisawa H., Sakai H., Yamasaki K., Wakayama T., Watanabe H., Otagiri M., Maruyama T. (2018). Biomimetic carbon monoxide delivery based on hemoglobin vesicles ameliorates acute pancreatitis in mice via the regulation of macrophage and neutrophil activity. Drug Deliv..

[19] Cabrales P., Tsai A.G., Intaglietta M. (2007). Hemorrhagic shock resuscitation with carbon monoxide saturated blood. Resuscitation.

[20] Motterlini R., Mann B.E., Foresti R. (2005). Therapeutic applications of carbon monoxide-releasing molecules. Expert Opin. Investig. Drugs.

[21] Motterlini R., Sawle P., Hammad J., Bains S., Alberto R., Foresti R., Green C.J. (2005). CORM-A1: a new pharmacologically active carbon monoxide-releasing molecule. FASEB J..

[22] Nikolic I., Saksida T., Vujicic M., Stojanovic I., Stosic-Grujicic S. (2015). Anti-diabetic actions of carbon monoxide-releasing molecule (CORM)-A1: immunomodulation and regeneration of islet beta cells. Immunol. Lett..

[23] Varadi J., Lekli I., Juhasz B., Bacskay I., Szabo G., Gesztelyi R., Szendrei L., Varga E., Bak I., Foresti R. (2007). Beneficial effects of carbon monoxide-releasing molecules on post-ischemic myocardial recovery. Life Sci..

[24] Fagone P., Mangano K., Mammana S., Cavalli E., Di Marco R., Barcellona M.L., Salvatorelli L., Magro G., Nicoletti F. (2015). Carbon monoxide-releasing molecule-A1 (CORM-A1) improves clinical signs of experimental autoimmune uveoretinitis (EAU) in rats. Clin. Immunol..

[25] Upadhyay K.K., Jadeja R.N., Thadani J.M., Joshi A., Vohra A., Mevada V., Patel R., Khurana S., Devkar R.V. (2018). Carbon monoxide releasing molecule A-1 attenuates acetaminophen-mediated hepatotoxicity and improves survival of mice by induction of Nrf2 and related genes. Toxicol. Appl. Pharmacol..

[26] Love S., Mudasir M.A., Bhardwaj S.C., Singh G., Tasduq S.A. (2017). Long-term administration of tacrolimus and everolimus prevents high cholesterol-high fructose-induced steatosis in C57BL/6J mice by inhibiting de-novo lipogenesis. Oncotarget.

[27] Liang W., Menke A.L., Driessen A., Koek G.H., Lindeman J.H., Stoop R., Havekes L.M., Kleemann R., van den Hoek A.M. (2014). Establishment of a general NAFLD scoring system for rodent models and comparison to human liver pathology. PLoS One.

[28] Cousin S.P., Hügl S.R., Wrede C.E., Kajio H., Myers M.G., Rhodes C.J. (2001). Free fatty acid-induced inhibition of glucose and insulin-like growth factor I-induced deoxyribonucleic acid synthesis in the pancreatic β-cell line INS-1. Endocrinology.

[29] Jana S., Patel D., Patel S., Upadhyay K., Thadani J., Mandal R., Das S., Devkar R. (2017). Anthocyanin rich extract of Brassica oleracea L. alleviates experimentally induced myocardial infarction. PLoS One.

[30] Scarpulla R.C. (2011). Metabolic control of mitochondrial biogenesis through the PGC-1 family regulatory network. Biochim. Biophys. Acta Mol. Cell Res..

[31] Ricchi M., Odoardi M.R., Carulli L., Anzivino C., Ballestri S., Pinetti A., Fantoni L.I., Marra F., Bertolotti M., Banni S. (2009). Differential effect of oleic and palmitic acid on lipid accumulation and apoptosis in cultured hepatocytes. J. Gastroenterol. Hepatol..

[32] García-Ruiz I., Solís-Muñoz P., Fernández-Moreira D., Muñoz-Yagüe T., Solís-Herruzo J.A. (2015). In vitro treatment of HepG2 cells with saturated fatty acids reproduces mitochondrial dysfunction found in nonalcoholic steatohepatitis. Dis. Model. Mech..

[33] Pérez-Carreras M., Del Hoyo P., Martın M.A., Rubio J.C., Martın A., Castellano G., Colina F., Arenas J., Solis-Herruzo J.A. (2003). Defective hepatic mitochondrial respiratory chain in patients with nonalcoholic steatohepatitis. Hepatology.

[34] Iuso A., Repp B., Biagosch C., Terrile C., Prokisch H. (2017). Assessing Mitochondrial Bioenergetics in Isolated Mitochondria from Various Mouse Tissues Using Seahorse XF96 Analyzer.

[35] Estes C., Razavi H., Loomba R., Younossi Z., Sanyal A.J. (2018). Modeling the epidemic of nonalcoholic fatty liver disease demonstrates an exponential increase in burden of disease. Hepatology.

[36] Mells J.E., Fu P.P., Sharma S., Olson D., Cheng L., Handy J.A., Saxena N.K., Sorescu D., Anania F.A. (2011). Glp-1 analog, liraglutide, ameliorates hepatic steatosis and cardiac hypertrophy in C57BL/6J mice fed a Western diet. Am. J. Physiol. Gastrointest. Liver Physiol..

[37] Lanaspa M.A., Sanchez-Lozada L.G., Choi Y.-J., Cicerchi C., Kanbay M., Roncal-Jimenez C.A., Ishimoto T., Li N., Marek G., Duranay M. (2012). Uric Acid induces hepatic steatosis by generation of mitochondrial oxidative stress potential role in fructose-dependent and-independent fatty liver. J. Biol. Chem..

[38] Sumida Y., Niki E., Naito Y., Yoshikawa T. (2013). Involvement of free radicals and oxidative stress in NAFLD/NASH. Free Radic. Res..

[39] Ribas V., García-Ruiz C., Fernández-Checa J.C. (2014). Glutathione and mitochondria. Front. Pharmacol..

[40] Kitteringham N.R., Abdullah A., Walsh J., Randle L., Jenkins R.E., Sison R., Goldring C.E., Powell H., Sanderson C., Williams S. (2010). Proteomic analysis of Nrf2 deficient transgenic mice reveals cellular defence and lipid metabolism as primary Nrf2-dependent pathways in the liver. J. Proteomics.

[41] Dinkova-Kostova A.T., Abramov A.Y. (2015). The emerging role of Nrf2 in mitochondrial function. Free Radic. Biol. Med..

[42] Kovac S., Angelova P.R., Holmström K.M., Zhang Y., Dinkova-Kostova A.T., Abramov A.Y. (2015). Nrf2 regulates ROS production by mitochondria and NADPH oxidase. Biochim. Biophys. Acta Gen. Subj..

[43] Piantadosi C.A., Carraway M.S., Babiker A., Suliman H.B. (2008). Heme oxygenase-1 regulates cardiac mitochondrial biogenesis via Nrf2-mediated transcriptional control of nuclear respiratory factor-1. Circ. Res..

[44] Pohjoismäki J.L., Wanrooij S., Hyvärinen A.K., Goffart S., Holt I.J., Spelbrink J.N., Jacobs H.T. (2006). Alterations to the expression level of mitochondrial transcription factor A, TFAM, modify the mode of mitochondrial DNA replication in cultured human cells. Nucleic Acids Res..

[45] Chang C.R., Blackstone C. (2010). Dynamic regulation of mitochondrial fission through modification of the dynamin‐related protein Drp1. Ann. N. Y. Acad. Sci..

[46] Nakamura S., Takamura T., Matsuzawa-Nagata N., Takayama H., Misu H., Noda H., Nabemoto S., Kurita S., Ota T., Ando H. (2009). Palmitate induces insulin resistance in H4IIEC3 hepatocytes through reactive oxygen species produced by mitochondria. J. Biol. Chem..

[47] Brookes P.S. (2005). Mitochondrial H+ leak and ROS generation: an odd couple. Free Radic. Biol. Med..

[48] Wallace D.C. (2012). Mitochondria and cancer. Nat. Rev. Cancer.

[49] Sandouka A., Balogun E., Foresti R., Mann B., Johnson T., Tayem Y., Green C., Fuller B., Motterlini R. (2005). Carbon monoxide-releasing molecules (CO-RMs) modulate respiration in isolated mitochondria. Cell. Mol. Biol..

[50] Braud L., Pini M., Muchova L., Manin S., Kitagishi H., Sawaki D., Czibik G., Ternacle J., Derumeaux G., Foresti R., Motterlini R. (2018). Carbon monoxide-induced metabolic switch in adipocytes improves insulin resistance in obese mice. JCI Insight.

[51] Iacono L.L., Boczkowski J., Zini R., Salouage I., Berdeaux A., Motterlini R., Morin D. (2011). A carbon monoxide-releasing molecule (CORM-3) uncouples mitochondrial respiration and modulates the production of reactive oxygen species. Free Radic. Biol. Med..

[52] Nishikawa T., Bellance N., Damm A., Bing H., Zhu Z., Handa K., Yovchev M.I., Sehgal V., Moss T.J., Oertel M. (2014). A switch in the source of ATP production and a loss in capacity to perform glycolysis are hallmarks of hepatocyte failure in advance liver disease. J. Hepatol..

