# Supplementary material for: Carbon monoxide releasing molecule-A1 improves nonalcoholic steatohepatitis via Nrf2 activation mediated improvement in oxidative stress and mitochondrial function
Source: Redox Biol. 2019 Aug 31;28:101314. doi: 10.1016/j.redox.2019.101314 (PMC6737302; doi:10.1016/j.redox.2019.101314)
Supplement: Multimedia component 1 [file mmc1.docx]

**Table 1 List of Primers for Real-time PCR**

**For Mice**

| Target | Primer | Sequence (5’🡪 3’) |
| --- | --- | --- |
| HO-1 | FP | ACATCGACAGCCCCACCAAGTTCAA |
|  | RP | CTGACGAAGTGACGCCATCTGTGAG |
| Nrf2 | FP | CGAGATATACGCAGGAGAGGTAAGA |
|  | RP | GCTCGACAATGTTCTCCAGCTT |
| GCLC | FP | AACACAGACCCAACCCAGAG |
|  | RP | CCGCATCTTCTGGAAATGTT |
| GCLM | FP | GCCCGCTCGCCATCTCTC |
|  | RP | GTTGAGCAGGTTCCCGGTCT |
| NQO1 | FP | CAGATCCTGGAAGGATGGAA |
|  | RP | TCTGGTTGTCAGCTGGAATG |
| NRF-1 | FP | GCAGAGGTGCAATCAAATGGA |
|  | RP | ATCGGTGGCGTTTCTCACTC |
| PGC-1α | FP | AGTCTTCGGCTGTTTGGTGA |
|  | RP | TGGAAGAACAGATGTGCCCC |
| SIRT1 | FP | GATACCTTGGAGCAGGTTGC |
|  | RP | CTCCACGAACAGCTTCACAA |
| FAS | FP | GGAGGTGGTGATAGCCGGTAT |
|  | RP | TGGGTAATCCATAGAGCCCAG |
| TFAM | FP | GGTCGCATCCCCTCGTCTA |
|  | RP | CCCCTGCCATGTGTTCTCCT |
| PPARα | FP | TGCAAACTTGGACTTGAACG |
|  | RP | TGATGTCACAGAACGGCTTC |
| CD36 | FP | TGAATGGTTGAGACCCCGTG |
|  | RP | TAGAACAGCTTGCTTGCCCA |
| Drp1 | FP | GGGCACTTAAATTGGGCTCC |
|  | RP | TGTATTCTGTTGGCGTGGAAC |
| SREBP1c | FP | GCAGCCACCATCTAGCCTG |
|  | RP | CAGCAGTGAGTCTGCCTTGAT |
| CPT-1 | FP | CGATCATCATGACTATGCGCTACT |
|  | RP | GCCGTGCTCTGCAAACATC |
| GAPDH | FP | TGTGAACGGATTTGGCCGTA |
|  | RP | ACTGTGCCGTTGAATTTGCC |
| Cytochrome b | FP | CCACTTCATCTTACCATTTA |
|  | RP | ATCTGCATCTGAGTTTAATC |
| Nuclear 18s rRNA | FP | GGGAGCCTGAGAAACGGC |
|  | RP | GGGTCGGGAGTGGGTAATTT |
| TNF-α | FP | GTGGAACTGGCAGAAGAG |
|  | RP | AATGAGAAGAGGCTGAGAC |
| IL-1β | FP | TCTATACCTGTCCTGTGTAATG |
|  | RP | GCTTGTGCTCTGCTTGTG |
| IL-6 | FP | TGGATGCTACCAAACTGGAT |
|  | RP | CCTCAAAGCCAAGATGAGAA |

**For Human**

| Target | Primer | Sequence (5’🡪3’) |
| --- | --- | --- |
| HO-1 | FP | TCTTGGCTGGCTTCCTTACC |
|  | RP | GGATGTGCTTTTCGTTGGGG |
| Nrf2 | FP | CTGCCAACTACTCCCAGGTT |
|  | RP | TGACTGAAACGTAGCCGAAGA |
| GCLC | FP | AGATTAGGCTGTCCTGGGTT |
|  | RP | TAAGGTACTGAAGCGAGGGT |
| GCLM | FP | GCGAGGAGGAGTTTCCAGATG |
|  | RP | CTGTGCAACTCCAAGGACTGA |
| NQO-1 | FP | GTTTGGAGTCCCTGCCATTCT |
|  | RP | GCAGAGAGTACATGGAGCCAC |
| NRF-1 | FP | TGGAACAAAATTGGGCCACG |
|  | RP | TGCCACCACCTGTTAAGCG |
| PGC-1α | FP | AGTTCACTCTCAGTAAGGGGC |
|  | RP | CCAGCTCCTGAATGACGCC |
| TFAM | FP | ACCAAAAAGACCTCGTTCAGC |
|  | RP | CGAGTTTCGTCCTCTTTAGCA |
| Drp1 | FP | CAAAGCAGTTTGCCTGTGGA |
|  | RP | TCTTGGAGGACTATGGCAGC |
| GAPDH | FP | GAGTCAACGGATTTGGTC |
|  | RP | GACAAGCTTCCCGTTCTC |
| Cytochrome b | FP | GCCCTCGGCTTACTTCTCTT |
|  | RP | AGTGATTGGCTTAGTGGGCG |
| Nuclear 18s rRNA | FP | ACGGACCAGAGCGAAAGCA |
|  | RP | GACATCTAAGGGCATCACAGAC |

FP, Forward Primer; RP, Reverse Primer
